# Fecal microbiota transplantation in severe pneumonia: a case report on overcoming pan-drug resistant *Klebsiella pneumoniae* infection

**DOI:** 10.3389/fmed.2024.1451751

**Published:** 2024-12-24

**Authors:** Liying Zhuang, Yanjing You, Shenyuan Zeng, Zongyang Yu, Huijuan Wang, Meiyan Chen, Wen Wen

**Affiliations:** Department of Respiratory and Critical Care Medicine, Fuzong Clinical Medical College of Fujian Medical University, Dongfang Hospital of Xiamen University, The 900th Hospital of Joint Logistics Support Force, Fuzhou, China

**Keywords:** fecal microbiota, fecal microbiota transplantation, severe pneumonia, pan-resistant bacterium, *Klebsiella pneumoniae*, Case report

## Abstract

**Objective:**

To evaluate the therapeutic potential of fecal microbiota transplantation (FMT) in treating severe pneumonia patients with concurrent pan-drug resistant *Klebsiella pneumoniae* infection.

**Methods:**

A case report of a 95-year-old female patient with severe pneumonia, complicated by pan-resistant bacterial infections, is presented. The patient was diagnosed with severe pneumonia caused by COVID-19, along with co-infections of *Staphylococcus hominis*, *Enterococcus faecalis*, *Candida tropicalis*, *Pseudomonas aeruginosa*, ESBL-producing pan-drug resistant *Klebsiella pneumoniae* and pan-resistant *Acinetobacter baumannii*. During hospitalization, the patient underwent comprehensive treatments, including antimicrobials, mechanical ventilation, and fiberoptic bronchoscopic alveolar lavage. FMT was administered following the failure of conventional treatments to resolve recurrent diarrhea, increased sputum production, and persistent pan-drug resistant *Klebsiella pneumoniae* infection.

**Results:**

Post-FMT, the patient exhibited significant clinical improvement, including reduced sputum production, cessation of diarrhea, and the normalization of respiratory symptoms. Gut microbiota analysis revealed that FMT enhanced the abundance of beneficial microbiota and suppressed *Klebsiella pneumoniae*, and the patient was successfully discharged after 133 days of hospitalization.

**Conclusion:**

FMT emerged as a pivotal intervention in the management of this severe pneumonia case, suggesting its efficacy in restoring gut microbiota balance and aiding recovery from multi-drug-resistant infections. This case underscores the potential of FMT as a therapeutic option in severe pulmonary infections, especially in the context of antibiotic resistance in severe pneumonia patients.

## 1 Case presentation

A 95-year-old female patient was admitted to the hospital with symptoms of fever, cough, and wheezing lasting for 1 day. On the morning of admission, she developed a fever without apparent cause, accompanied by a cough, white sputum, wheezing and fatigue. An emergency department visit confirmed a positive COVID-19 antigen test. Upon admission, her vital signs were as follows: temperature 38.1°C, heart rate 70 beats per minute, respiratory rate 28 breaths per minute, and blood pressure 158/77 mmHg. She was conscious and in acute distress, with a painful expression and semi-reclined posture. Coarse breath sounds and scattered wet rales were heard in both lungs, without any dry rales or pleural friction rub. No significant abnormalities were noted in the rest of the cardiovascular and abdominal examination. Past medical history included hypertension for over 30 years, controlled poorly with Nifedipine sustained-release tablets (30 mg daily), a 25-year history of diabetes mellitus controlled with Metformin, and a 10-year history of Parkinson’s disease. She was also diagnosed with osteoporosis and thoracolumbar vertebral compression fractures over a year ago.

**Figure 1:**
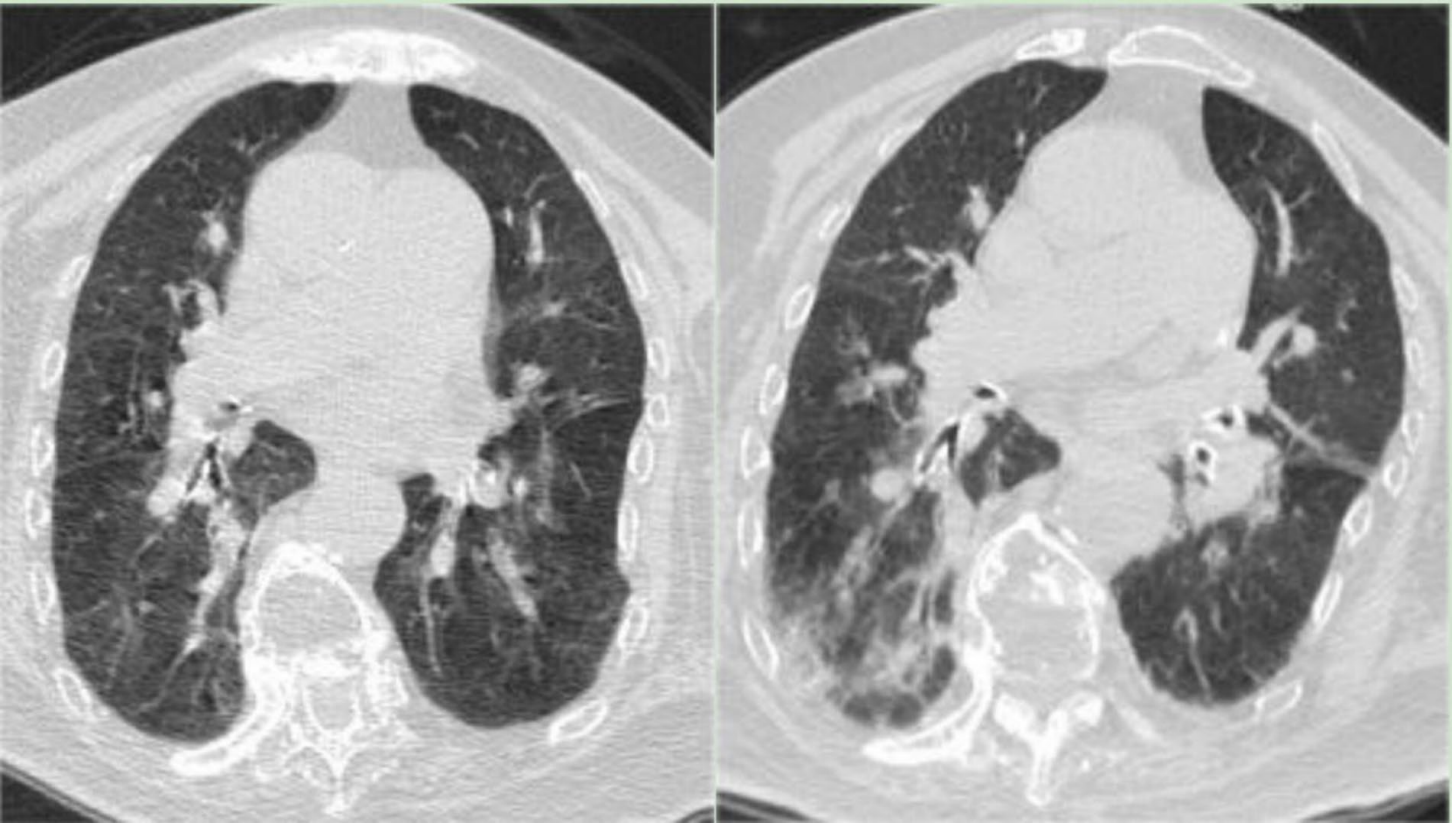
(Left) Chest CT on December 29, 2022, indicating bilateral bronchiolitis. (Right) Chest CT on January 3, 2023, showing scattered patchy high-density shadows in both lungs, suggestive of inflammatory lesions.

Post-admission monitoring indicated fluctuating blood oxygen saturation levels between 88 and 93%. Blood gas analysis showed a reduced oxygen partial pressure of 63.2 mmHg and a carbon dioxide partial pressure of 41.9 mmHg. Blood tests revealed a lymphocyte count of 0.43*10^9/L, normal granulocyte count, normal C-reactive protein (CRP) and procalcitonin (PCT) levels, and no significant abnormalities in biochemistry. Chest CT scans showed bilateral bronchiolitis (Figure 1, Left). After 6 days of treatment with Ceftriaxone, there was no significant improvement in symptoms, with body temperature fluctuating between 37.1–38.2°C, declining spirits, and decreasing blood oxygen levels. A follow-up blood gas analysis showed a pH of 7.345, an oxygen partial pressure of 52.2 mmHg, and a carbon dioxide partial pressure of 60.0 mmHg. Blood tests indicated a lymphocyte count of 0.62*10^9/L, an elevated CRP level of 66.3 mg/L, and normal PCT; the chest CT showed scattered patchy high-density shadows in both lungs, suggestive of inflammatory lesions (Figure 1, Right); Consultations with respiratory and critical care medicine suggested severe pneumonia, and treatment with methylprednisolone for inflammation, combined with Ceftriaxone, Moxifloxacin for infection, Azvudine for antiviral therapy, and Thymalfasin to boost immunity, showed unsatisfactory results. The patient experienced worsening asthma and coughing up phlegm, with progressively declining blood oxygen levels. Bedside fiberoptic bronchoscopy for sputum aspiration revealed tracheal softening and collapse with white mucous sputum; she was then transferred to the intensive care unit for continued treatment, receiving orotracheal intubation, mechanical ventilation, and enteral nutrition support. Blood tests revealed a lymphocyte count of 0.19*10^9/L, accompanied by a normal white blood cell (WBC) count, a normal granulocyte count, and CRP of 53.3 mg/L; Blood cultures indicated *Staphylococcus hominis*, prompting a switch to Teicoplanin and Cefoperazone Sulbactam for infection treatment.

A repeat COVID-19 nucleic acid test remained positive (Ct value 24.33) after 12 days, leading to a switch to Paxlovid for antiviral treatment and prone ventilation. Every other day, fiberoptic bronchoscope alveolar lavage was performed; BALF mNGS results showed *Enterococcus faecium* and *Candida tropicalis*, prompting the addition of Micafungin for infection treatment. Due to symptom improvement and CRP reduction, the patient discontinued Teicoplanin and Cefoperazone Sulbactam and switched to Meropenem for infection treatment, subsequently Paxlovid and methylprednisolone were also discontinued. Inflammation markers increased compared to previous readings after 20 days, blood test showed white blood cell count of 13.92*10^9/L, granulocyte count of 13.39*10^9/L, lymphocyte count of 0.30*10^9/L, and CRP of 252 mg/L. Then a follow-up BALF culture indicated *Candida tropicalis* + *Acinetobacter baumannii complex/Acinetobacter haemolytica*. The anti-infection treatment plan was adjusted to a combination therapy based on Voriconazole, Tigecycline, Polymyxin B, and intravenous immunoglobulin, leading to an improvement in the patient’s condition. A follow-up chest x-ray after 31 days of hospitalization showed bilateral pneumonia, demonstrating improvement in the left lung while indicating slight progression in the right lung, accompanied by a minor accumulation of pleural effusion on the left side. The patient still had a fever, PCT rises to 1.05 ng/mL, and BALF culture indicated *Pseudomonas aeruginosa* infection.

**Figure 2:**
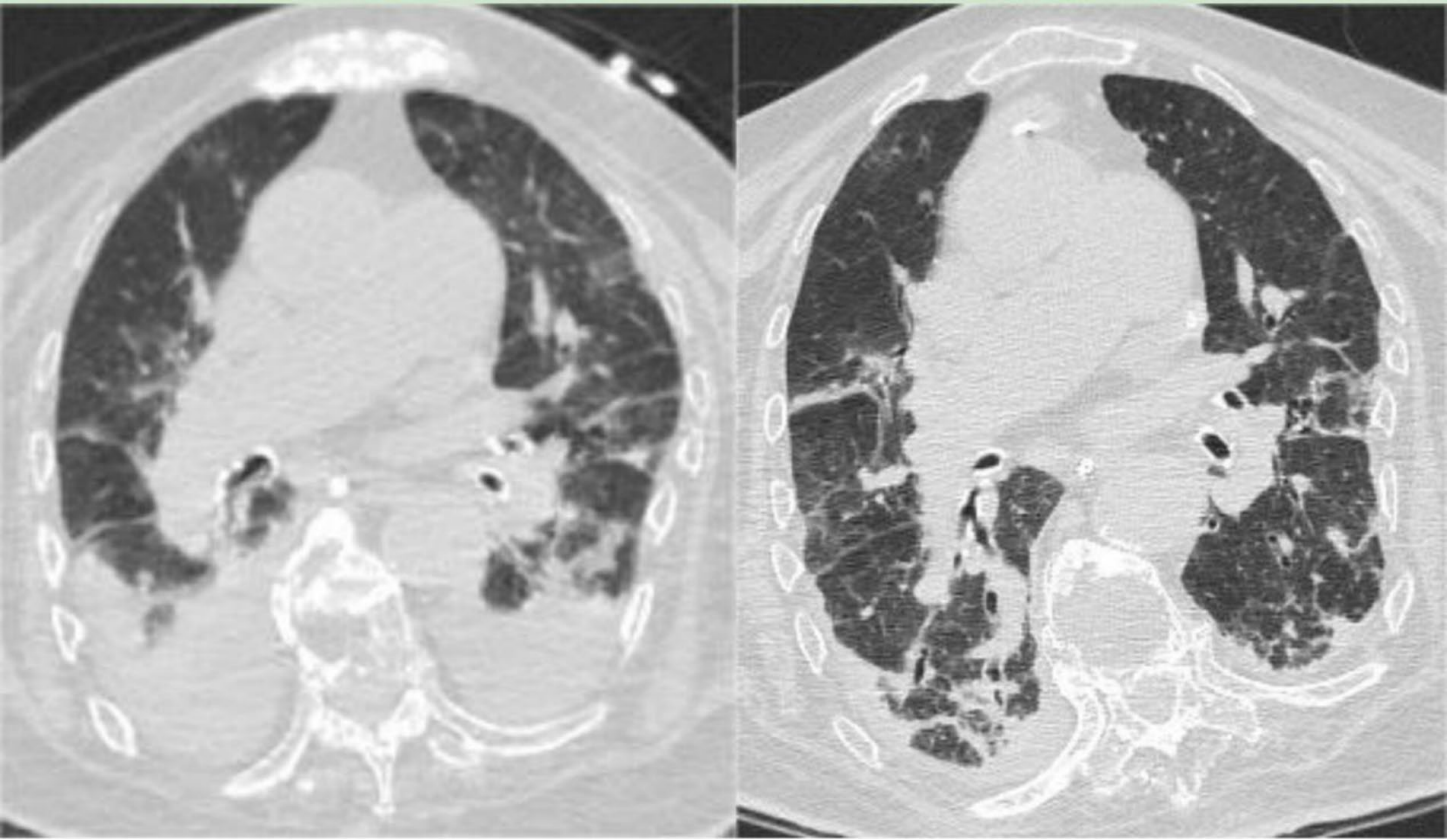
(Left) Chest CT on February 1, 2023, indicating bilateral pneumonia with bilateral pleural effusion and bilateral lower lung atelectasis, showing improvement from the previous scan (January 3, 2023). (Right) Chest CT on March 23, 2023, showing bilateral pneumonia with bilateral pleural effusion and bilateral lower lung atelectasis, improved compared to the February 1, 2023, CT scan.

After 34 days, the tracheal tube was removed, and the patient was switched to high-flow nasal cannula oxygen therapy, showing improved mental status. A follow-up chest CT on the same day showed bilateral pneumonia with bilateral pleural effusion and bilateral lower lung atelectasis, improved compared to the CT scan on the sixth day of hospitalization (Figure 2, Left). After 36 days, the patient was stepped down to Levofloxacin combined with Piperacillin Sodium Tazobactam for infection treatment. The patient resumed enteral nutrition after 38 days but subsequently developed intestinal dysbiosis, initially presenting as constipation. Stool culture indicated *Bacillus cereus* and Mould presence. BALF mNGS results showed *Acinetobacter baumannii* and *Corynebacterium striatum*, with BALF culture growing *Sphingomonas paucimobilis*. Based on drug sensitivity, Piperacillin Sodium Tazobactam was discontinued and replaced with Tigecycline, continuing in combination with Levofloxacin. On the third day after the occurrence of intestinal flora dysbiosis, a T lymphocyte subset test was performed, showing an absolute count of CD4 cells of 248 cells per microliter, indicating that the patient experienced immune dysfunction. The patient intermittently retested for COVID-19 nucleic acid test until it turned negative on the 39th day of hospitalization. On the 51st day, with WBC and PCT levels normalized, Tigecycline and Levofloxacin were discontinued, and the patient was stepped down to Piperacillin Sodium Tazobactam for infection treatment, and transferred to a general respiratory ward in three more days. On the 58th day, a follow-up BALF mNGS revealed *Klebsiella pneumoniae*, *Acinetobacter baumannii*, *Corynebacterium striatum*, *Enterococcus faecium*, and *Candida albicans*, with BALF culture growing ESBL-producing, pan-drug resistant *K. pneumoniae* (PDR-KP) and pan-resistant *Acinetobacter baumannii*. Amikacin nebulization was added, Piperacillin Sodium Tazobactam was discontinued, and Micafungin + Teicoplanin + Isepamicin was started, with daily bronchoscopy. On the 63rd day, a follow-up BALF culture showed PDR-KP; Teicoplanin was discontinued and replaced with Tigecycline, continuing in combination with Micafungin and Isepamicin for infection treatment. Due to Significant inflammatory alterations and increased secretions in the airway as seen in bronchoscopy, Polymyxin E was added to the bronchoscopic treatment after 62 days.

**Figure 3:**
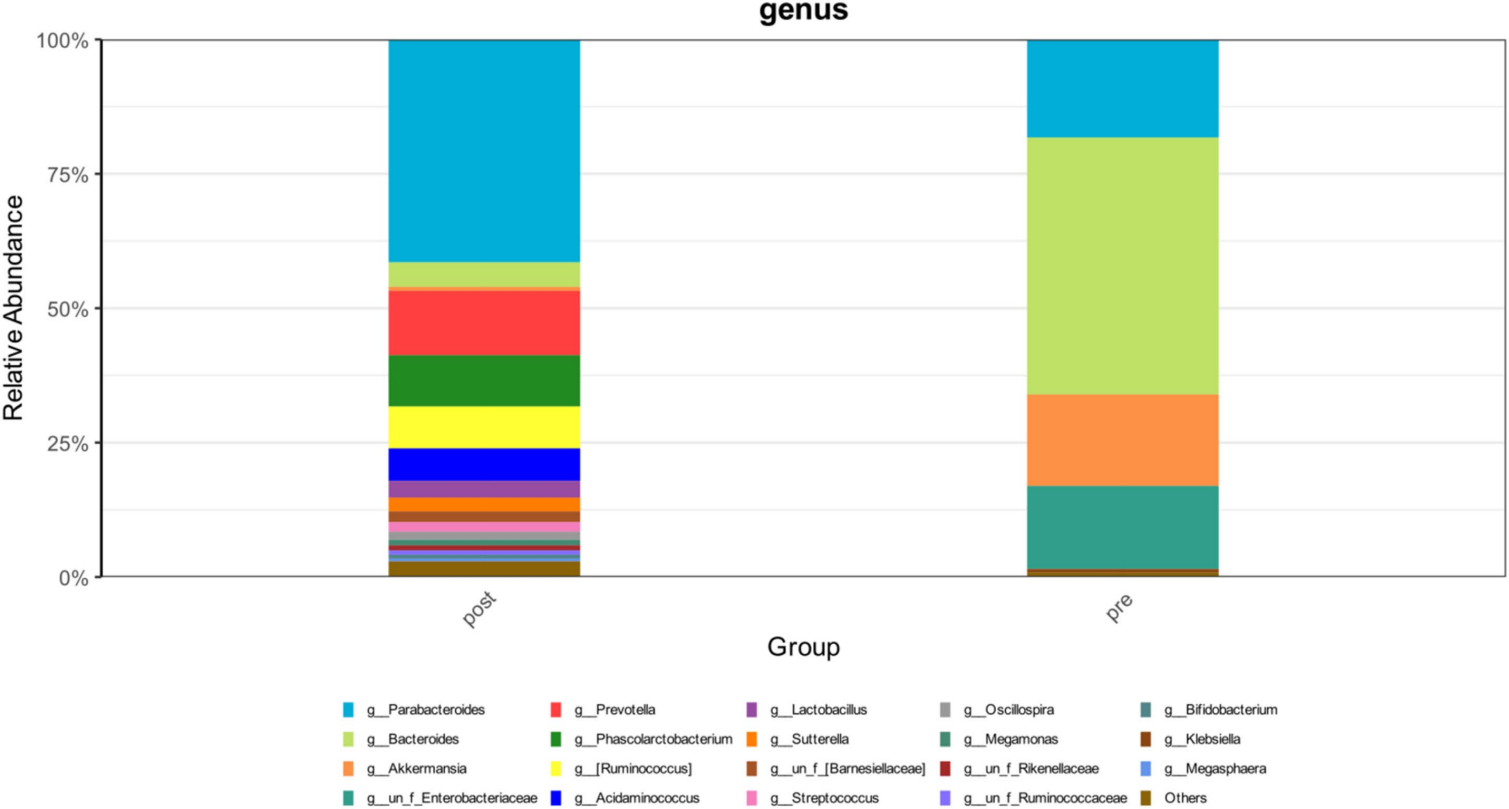
The changes in the abundance distribution of the patient’s intestinal microbiota before and after FMT.

**Figure 4:**
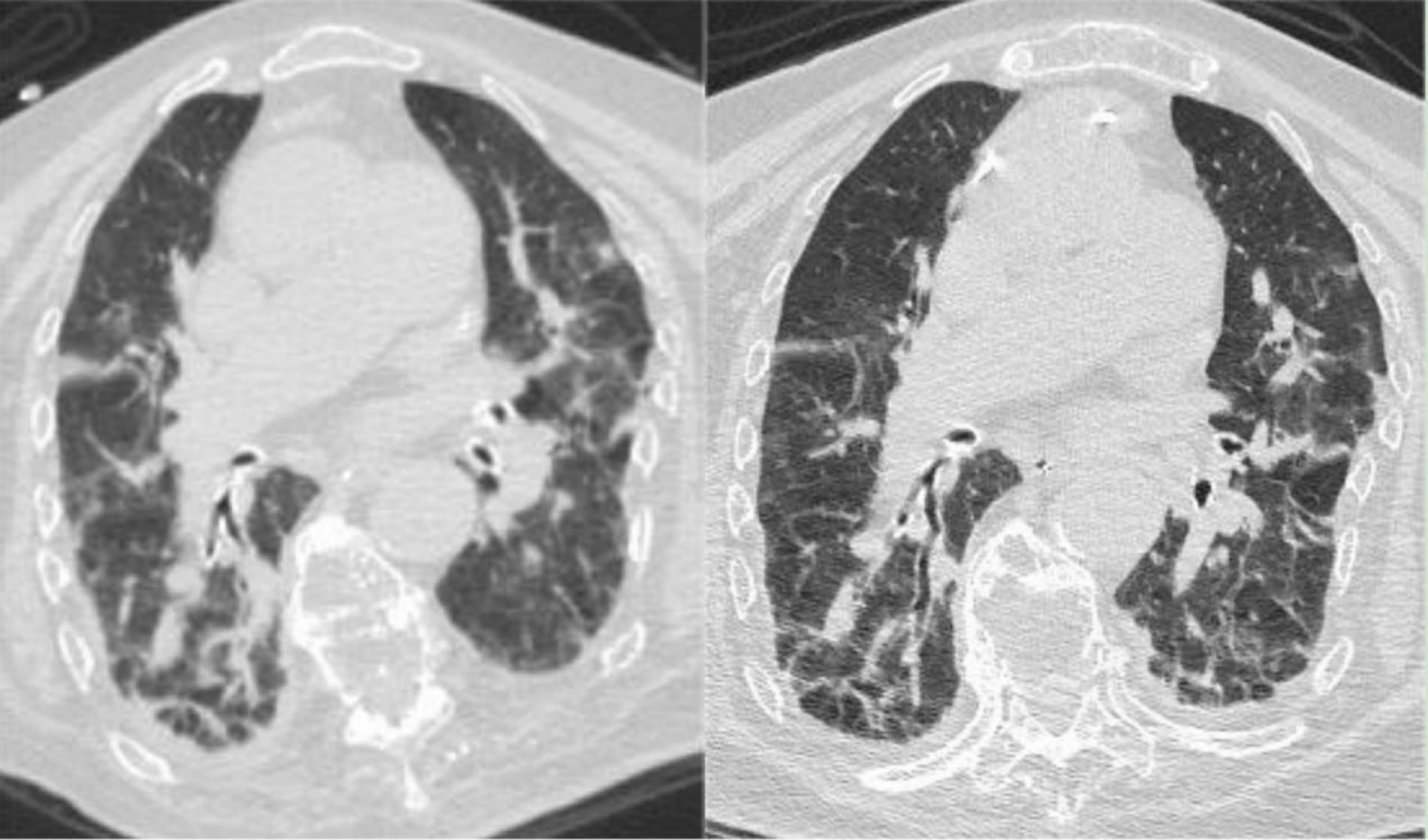
(Left) Chest CT on April 18, 2023, indicating bilateral pneumonia with bilateral pleural effusion and bilateral lower lung atelectasis, improved compared to the March 23, 2023, CT scan. (Right) Chest CT on May 8, 2023, showing bilateral pneumonia with bilateral pleural effusion, improved compared to the April 18, 2023, CT scan.

During the treatment period, the patient experienced recurrent diarrhea from the 50th day of hospitalization, symptomatic treatment for diarrhea and regulation of the intestinal microbiota showed poor results. Given the potential for significant intestinal dysbiosis, we employed 16S rRNA gene-based molecular methodologies to delineate the intestinal microbiota’s composition in the patient. Intestinal microbiota testing revealed a diminished gut microbiota diversity, abnormal nutritional synthesis metabolism, a heightened abundance of Klebsiella, and a notable depletion of beneficial bacteria, particularly *Prevotella*, *Phascolarctobacterium*, *Rumimococcus*, *Acidaminococcus*, and *Lactobacillus* (Figure 3). The discovery prompted the determination to rectify the imbalance through fecal microbiota transplantation. We administered a healthy donor fecal suspension (1) via nasojejunal tube at 150 mL per injection on the 68th day of hospitalization, a total of 4 times over 6 days. After the first transplantation, the patient’s diarrhea ceased, and the secretion in the tracheal cavity significantly reduced, leading to the discontinuation of Micafungin. All antimicrobial drugs were discontinued on the 73rd day, with bronchoscopy reduced to once every 2 days. After a complete course of four treatments, the patient showed significant improvement, with multiple stool tests and cultures negative. Analysis of gut microbiota composition on day 27 post-FMT demonstrated an increase in beneficial microbiota and a concurrent reduction in the relative abundance of the opportunistic pathogen *K. pneumoniae* (Figure 3). The patient was then maintained on probiotics to regulate the intestinal microbiota. The patient then underwent multiple CT scan, all of which showed improvement compared with the previous CT scan (Figures 2, 4, Right).On the 122nd day, we repeated BALF culture was negative. The patient was successfully discharged after a 133-day hospital stay (Supplementary Table 1 for more detailed inflammation markers record and clinical course event).

## 2 Discussion

Patients with severe pneumonia often have underlying diseases and are more likely to experience immune cell exhaustion, resulting in lower immune function than patients with milder forms of the disease. Furthermore, treatment methods such as prolonged use of corticosteroids, antibiotics, and invasive ventilation are often a must due to serious illness, which can impair the body’s immune function, increasing the likelihood of secondary infections, including multi-drug-resistant bacterial infections. In China, severe pneumonia is often combined with *Pseudomonas aeruginosa* and *K. pneumoniae* infection for adults (2). And for severe COVID-19 patients, the prevalent pathogens are often hospital-acquired strains such as *Acinetobacter baumannii* and *K. pneumoniae*, which are highly resistant to antibiotics (3). Invasive fungal infections should not be overlooked in severe pneumonia patients. Especially for mechanically ventilated severe COVID-19 patients, the incidence of invasive pulmonary aspergillosis and Trichosporon infections has risen (4). Surveys indicate an increase in fungal infection-related deaths during 2020–2021, primarily involving *Aspergillus* and *Trichosporon* species, with secondary infections linked to life-threatening complications (acute kidney injury, acute respiratory distress syndrome) and increased mortality rates (5).

In our case, the initial BALF mNGS test was negative. After transfer to the ICU and intubation, blood cultures indicated *Staphylococcus hominis*. During her stay, repeated BALF mNGS tests revealed *Enterococcus faecalis*, *Candida tropicalis*, *Pseudomonas aeruginosa*, ESBL-producing PDR-KP and pan-resistant *Acinetobacter baumannii*. The patient required corticosteroids, prolonged combined antibiotic therapy, and invasive ventilation. She developed multiple bacterial and fungal infections, including two pan-resistant strains.

Treating severe pneumonia patients caused by COVID-19 with combined bacterial and fungal infections inevitably involves long-term use of multiple antimicrobials. However, this can complicate treatment by:

1. Leading to infections by multi-drug and pan-resistant bacteria: Antibiotic resistance is common among bacterial co-infections or secondary infections in COVID-19 patients (6). Studies show that using ≥4 types of antibiotics is an independent risk factor for multi-drug resistant bacterial infections in critical COVID-19 cases (7). Overuse of empirical antifungal treatments can also increase resistance. This prolongs hospital and ICU stays, increases costs and mortality rates, and raises the risk of adverse reactions to antimicrobials (8).
2. Disrupting gut microbiota: The gut microbiota, comprising billions of microbes, plays a crucial role in human health. Prolonged (9) and combined (10) antibiotic use disrupts this dynamic community, leading to increased inflammation, disturbed gut immune homeostasis, and heightened susceptibility to infections (11). COVID-19 itself can induce dysbiosis, characterized by the overgrowth of opportunistic pathogens, which can lead to secondary bloodstream infections (12). Gut dysbiosis also increases susceptibility to respiratory diseases due to changes in immune responses and lung homeostasis (gut-lung axis) (13) and adversely affects brain function and cognition, linked to changes in immune cell function, activation of microglia, and reduced cholinergic gamma oscillations in the hippocampus (14).

In our case, the patient developed severe intestinal dysbiosis on the 44th day of prolonged combined antibiotic use, leading to recurrent diarrhea. Intestinal microbiota testing indicates a reduction in the abundance of beneficial bacteria, while conditional pathogens, such as *K. pneumoniae*, exhibit overgrowth. Standard antidiarrheal and probiotic treatments failed to correct the dysbiosis, and severe gastrointestinal dysfunction affected her immune function. Additionally, recurrent diarrhea prevented adequate nutritional support, hindering immune recovery and infection control. To overcome this challenge, fecal microbiota transplantation (FMT) was attempted after thorough communication with the patient’s family.

FMT, which involves transferring pre-screened fecal matter from a healthy donor to the patient’s gastrointestinal tract, initially recommended for recurrent *Clostridium difficile* infections (15). For recurrent *Clostridium difficile*, antibiotic treatment can exacerbate dysbiosis, while FMT can correct it and reduce the incidence of adverse events and all-cause mortality (16).

In recent years, FMT has been increasingly applied in other areas. Beyond gastrointestinal diseases (like ulcerative colitis (17), irritable bowel syndrome (18), etc.), gut microbiota has been linked to the efficacy of anti-PD-1 treatment in melanoma, renal cell carcinoma, and non-small cell lung cancer. FMT alters the gut microbiota and reprograms the tumor microenvironment to overcome resistance to anti-PD-1 immunotherapy (19). Gut dysbiosis is also associated with the onset and progression of neurological disorders. As an intervention related to the gut microbiota, FMT can be used to treat neurological diseases (including autism spectrum disorders, Parkinson’s disease, schizophrenia, etc.) (20). Additionally, FMT can improve insulin sensitivity in patients with severe obesity and metabolic syndrome (21).

A review on the therapeutic efficacy of FMT across diseases (22) showed that FMT is effective in treating 85 diseases across eight categories (including infections, gastrointestinal diseases, microbiome-gut-liver axis, microbiome-gut-brain axis, metabolic diseases, oncology, hematologic diseases, and others), with a few small-scale studies mentioning its application in COVID-19 and COPD. In the respiratory disease field, apart from the aforementioned applications in non-small cell lung cancer, COVID-19, and COPD, a prospective non-randomized controlled study (23) indicated that in severe pneumonia recovery, FMT application can mitigate the inflammatory response by reducing the abundance of harmful bacteria, rebuilding the gut microbiota structure, improving metabolism and function, and has a promising application in severe lung infections.

In our case, the severe pneumonia patient benefited greatly from FMT, showing significant symptom improvement after four treatments. The abundance of beneficial bacteria in the intestinal microbiota has returned, and the abundance of *K. pneumoniae* had decreased. Leading to continuous improvement and eventual discharge.

Notably, in the later stage, the patient was in a state of persistent PDR-KP infection. Comparative analysis of the gut microbiota before and after FMT revealed a complete elimination of *K. pneumoniae* from the intestinal microbiome, with its abundance reduced to undetectable levels. Furthermore, BALF cultures confirmed the absence of PDR-KP. These findings strongly suggest that FMT has the potential to suppress or even eradicate *K. pneumoniae* colonization and infection through microbiota modulation.

Short-chain fatty acids (SCFAs) are crucial and beneficial metabolic products of gut microbiota, possessing important physiological functions and maintaining immune homeostasis. The gut microbiota can intervene in *K. pneumoniae* pneumonia through SCFAs (24). The Metabolic Sensor GPR43 Receptor plays a vital role in this process (25). The restoration of SCFA-producing beneficial bacteria following FMT may be a key mechanism in suppressing *K. pneumoniae*. Additionally, healthy microbiota can limit pathogen survival and proliferation through competitive exclusion and nutrient competition.

Previous studies have demonstrated FMT’s effectiveness in antibiotic-resistant bacteria infection: decolonization of antibiotic-resistant bacteria in the gut (26), treatment of carbapenem-resistant *K. pneumoniae* (CRKP) infection in renal transplant patients (27), and eradication of carbapenem-resistant Enterobacteriaceae colonization (28). This study suggests that FMT could serve as a valuable adjunctive therapy for managing *K. pneumoniae* infections, particularly in cases involving antibiotic resistance. Large-scale, well-designed randomized controlled trials are needed to further explore the safety and clinical utility of this therapeutic approach.

## 3 Conclusion

Patients with severe pneumonia are prone to multiple bacterial and fungal co-infections. The prolonged use of broad-spectrum antibiotics often complicates treatment, leading not only to infections by multi-drug-resistant and even pan-resistant bacteria but also to significant dysbiosis of the gut microbiome and gastrointestinal dysfunction. This dysbiosis adversely affects immune function and impedes the patient’s ability to receive adequate nutrition, greatly impacting the treatment process. Fecal microbiota transplantation (FMT) can help rebuild the gut microbiota, regulate the airway microecology, and enhance the patient’s immunity, showing promising prospects in the treatment of severe pulmonary infections.

## Data Availability

The raw data supporting the conclusions of this article will be made available by the authors, without undue reservation.

## Supplementary material

SUPPLEMENTARY TABLE 1 Inflammation markers record and clinical course event during a 133-day hospital stay.
